# Identification of a low risk population for parametrial invasion in patients with early-stage cervical cancer

**DOI:** 10.1186/s12967-018-1531-6

**Published:** 2018-06-14

**Authors:** Yohann Dabi, Claire Willecocq, Marcos Ballester, Xavier Carcopino, Sofiane Bendifallah, Lobna Ouldamer, Vincent Lavoue, Geoffroy Canlorbe, Emilie Raimond, Charles Coutant, Olivier Graesslin, Pierre Collinet, Alexandre Bricou, Cyrille Huchon, Emile Daraï, Bassam Haddad, Cyril Touboul, For the Groupe de Recherche FRANCOGYN

**Affiliations:** 1 Department of Obstetrics and Gynecology, Centre Hospitalier Intercommunal, Créteil, France; 2 Faculté de Médecine de Créteil UPEC – Paris XII, Créteil, France; 3 Department of Gynaecology and Obstetrics, Tenon University Hospital, Assistance Publique des Hôpitaux de Paris (AP-HP), University Pierre and Marie Curie, Paris 6, Institut Universitaire de Cancérologie (IUC), Paris, France; 4 Department of Obstetrics and Gynecology, Hopital Nord, APHM, Marseilles, France; 5 Department of Obstetrics and Gynaecology, Centre Hospitalier Régional Universitaire de Tours, Hôpital Bretonneau, Tours, France; 6 CRLCC Eugène-Marquis, Service de Gynécologie, CHU de Rennes, Université de Rennes 1, Rennes, France; 7 Department of Gynaecology and Obstetrics, Pitié Salpetrière University Hospital, Assistance Publique des Hôpitaux de Paris (AP-HP), University Pierre and Marie Curie, Paris 6, Institut Universitaire de Cancérologie (IUC), Paris, France; 8 Department of Obstetrics and Gynaecology, Institute Alix de Champagne University Hospital, Reims, France; 9 Centre de lutte contre le cancer Georges François Leclerc, Dijon, France; 10 Department of Obstetrics and Gynecology, Centre Hospitalier Régional Universitaire, Lille, France; 11 Department of Obstetrics and Gynecology, Jean-Verdier University Hospital, Assistance Publique des Hôpitaux de Paris (AP-HP), Paris, France; 12 EA 7285 Research Unit “Risk and Safety in Clinical Medicine for Women and Perinatal Health”, Versailles-Saint-Quentin University (UVSQ), 78180 Montigny-le-Bretonneux, France; 13 Department of Gynaecology and Obstetrics, Intercommunal Hospital Centre of Poissy-Saint-Germain-en-Laye, 78103 Poissy, France; 14 Inserm U965 Laboratory, Angiogenèse et Recherche Translationnelle, Paris, France

**Keywords:** Cervical cancer, Early-stage, Radical surgery, Predicting, Parametrial invasion, Low-risk

## Abstract

**Background:**

Recent studies have challenged radical procedures for less extensive surgery in selected patients with early-stage cervical cancer at low risk of parametrial invasion. Our objective was to identify a subgroup of patients at low risk of parametrial invasion among women having undergone surgical treatment.

**Methods:**

Data of 1447 patients with cervical cancer treated between 1996 and 2016 were extracted from maintained databases of 10 French University hospitals. Patients with early-stage (IA2–IIA) disease treated by radical surgery including hysterectomy and trachelectomy, were selected for further analysis. The Kaplan–Meier method was used to estimate the survival distribution. A Cox proportional hazards model including all the parameters statistically significant in univariate analysis, was used to account for the influence of multiple variables.

**Results:**

Out of the 263 patients included for analysis, on final pathology analysis 28 (10.6%) had parametrial invasion and 235 (89.4%) did not. Factors significantly associated with parametrial invasion on multivariate analysis were: age > 65 years, tumor > 30 mm in diameter measured by MRI, lymphovascular space invasion (LVSI) on pathologic analysis. Among the 235 patients with negative pelvic lymph nodes, parametrial disease was seen in only 7.6% compared with 30.8% of those with positive pelvic nodes (p < 0.001). In a subgroup of patients presenting tumors < 30 mm, negative pelvic status and no LVSI, the risk of parametrial invasion fell to 0.6% (1/173 patients).

**Conclusion:**

Our analysis suggests that there is a subgroup of patients at very low risk of parametrial invasion, potentially eligible for less radical procedures.

**Electronic supplementary material:**

The online version of this article (10.1186/s12967-018-1531-6) contains supplementary material, which is available to authorized users.

## Background

The success of prevention campaigns and widespread screening for cervical cancer has led to more women being diagnosed with early-stage disease [1, 2]. Current recommendations for surgical management of patients with early-stage cervical cancer include modified radical hysterectomy and pelvic lymph node dissection (PLND). Women desiring to preserve fertility can be treated with radical trachelectomy associated with PLND [3, 4]. The rationale for “radical” surgery is the extent to which paracervical tissue is involved and the risk of lymphatic disease. Indeed, cervical cancer has a lymphatic spread pattern and the parametrium is a key area of the cervical plexus drainage area [5, 6].

Recent studies have challenged these radical procedures for less extensive surgery in selected patients [7, 8] with a low risk of parametrial invasion. This is important as extensive lymphadenectomy and parametrectomy are the main causes of postoperative complications [9–11]. Serious urinary and rectal dysfunction, impairing patients’ quality of life, have been reported after parametrial resection [12–15]. Analysis of the nerve and lymphatic pathways have shown that the technique of radical hysterectomy could change to spare high density nerve regions [16, 17], and recent studies have even suggested there might be a subgroup of patients that could be treated without radical surgery [18–21]. The crucial issue remains of which criteria could best select patients who might benefit from less radical surgery without jeopardizing oncological results.

The main objective of our study was to identify a subgroup of patients with a very low risk of parametrial invasion among women who had been surgically treated for early-stage cervical cancer.

## Methods

### Patients included

We conducted a retrospective analysis of patients treated in 10 French institutions (Creteil University hospital, Tenon University Hospital, Reims University Hospital, Dijon cancer center, Lille University hospital and Lille cancer center, Tours University hospital, Bondy University hospital, Rennes University hospital, Marseille Public hospital North). The research protocol was approved by the Institutional Review Board (IRB) of the French College of Obstetrics and Gynaecology (CEROG 2016—GYN—0502).

The maintained databases included all patients treated with radical surgery (i.e., trachelectomy or hysterectomy) for early-stage cervical cancer between January 1996 and December 2016. Early-stage cervical cancer was defined as disease stages IA2–IIA clinically, and on preoperative pelvic magnetic resonance imaging (MRI) according to the latest 2009 International Federation of Gynecology and Obstetrics (FIGO) classification [22].

The following patient characteristics were extracted from their medical charts: age, BMI, medical and surgical history, surgical procedure, FIGO stage, final pathological analysis, treatment received.

Inclusion criteria:

- Stage IA2–IIA, based on our review of the literature, as this choice was made by many reports including prospective studies evaluating less radical surgery in this subgroup of patients.
- Patients treated by type II–III hysterectomy as described by Piver-Rutledge-Smith in 1974 [23] or type B-C according to the Querleu and Morrow classification [24].
- Patients diagnosed with stage IA2–IB1 desiring to preserve their fertility, treated with radical trachelectomy in accordance with the latest European guidelines [3].

Exclusion criteria:

- Preoperative FIGO stage > IIA.
- Non-radical surgery, i.e., without parametrectomy.
- Preoperative brachytherapy.
- Patients with missing histologic data such as parametrial involvement, lymphovascular space invasion (LVSI), and the size of the tumor.

### Initial management

All patients underwent clinical examination. Disease stage was initially assessed by preoperative pelvic MRI including an evaluation of tumor size. Indeed, this exam is more powerful than clinical examination to precisely assess parametrial invasion, the presence of extra uterine disease and tumor size.

Patients with FIGO stage IA2–IIA2 underwent bilateral pelvic lymph node dissection and radical hysterectomy or radical trachelectomy. Patients with preoperative brachytherapy—if the tumor size was > 2 cm or if there was LVSI—were excluded. Similarly, patients with peroperative diagnosis of metastatic pelvic nodes were also excluded as they did not undergo radical hysterectomy but subsequent surgical paraaortic staging. Operative complications were evaluated using the Clavien–Dindo classification. Complications of grade III (requiring surgical, endoscopic or radiological intervention) or more were considered severe.

Parametrial invasion as well as the main tumor characteristics were determined by pathologic analysis. Parametrium involvement was defined as the presence of tumor cells in or beyond the parametrial vessels. Each of the participating centers conducted pathologic analysis according to local practice. None of the centers in our cohort performed ultra-staging.

The decision to perform adjuvant therapy, such as vaginal brachytherapy or concomitant chemoradiotherapy and brachytherapy, was made by multidisciplinary committees according to the national guidelines at the time the patient was treated [3].

Follow-up protocols included gynecological examination every 3 months for 2 years and then every 6 months for 2 years. Computed tomography (CT) or positron emission tomography—computed tomography (PET/CT) scans were performed systematically when clinically indicated. Recurrences were diagnosed either by biopsy or with an imaging exam. Disease-free survival (DFS) and overall survival (OS) were calculated from the date of the initial surgery.

### Statistical analysis

Databases were managed using Excel (Microsoft Corporation, Redmond, WA, USA) and statistical analyses were performed using R software (3.3.1 version, available online). Statistical analysis was based on the Student’s t test for continuous variable and the χ^2^ test or Fisher’s exact test for categorical variables. The Kaplan–Meier method was used to estimate the survival distribution. Comparisons of survival were made using the log rank test. A logistic regression model including all the parameters statistically significant in univariate analysis, was used to account for the influence of multiple variables. Values of p < 0.05 were considered to denote significant differences. Optimal cut off for age and size were obtained by a minimal p value approach. The performance of the model was quantified with respect to discrimination and calibration. An internal validation of the model was performed with a bootstrapping method to obtain relatively unbiased estimates.

## Results

### Main characteristics of the patients included

**Fig. 1:**
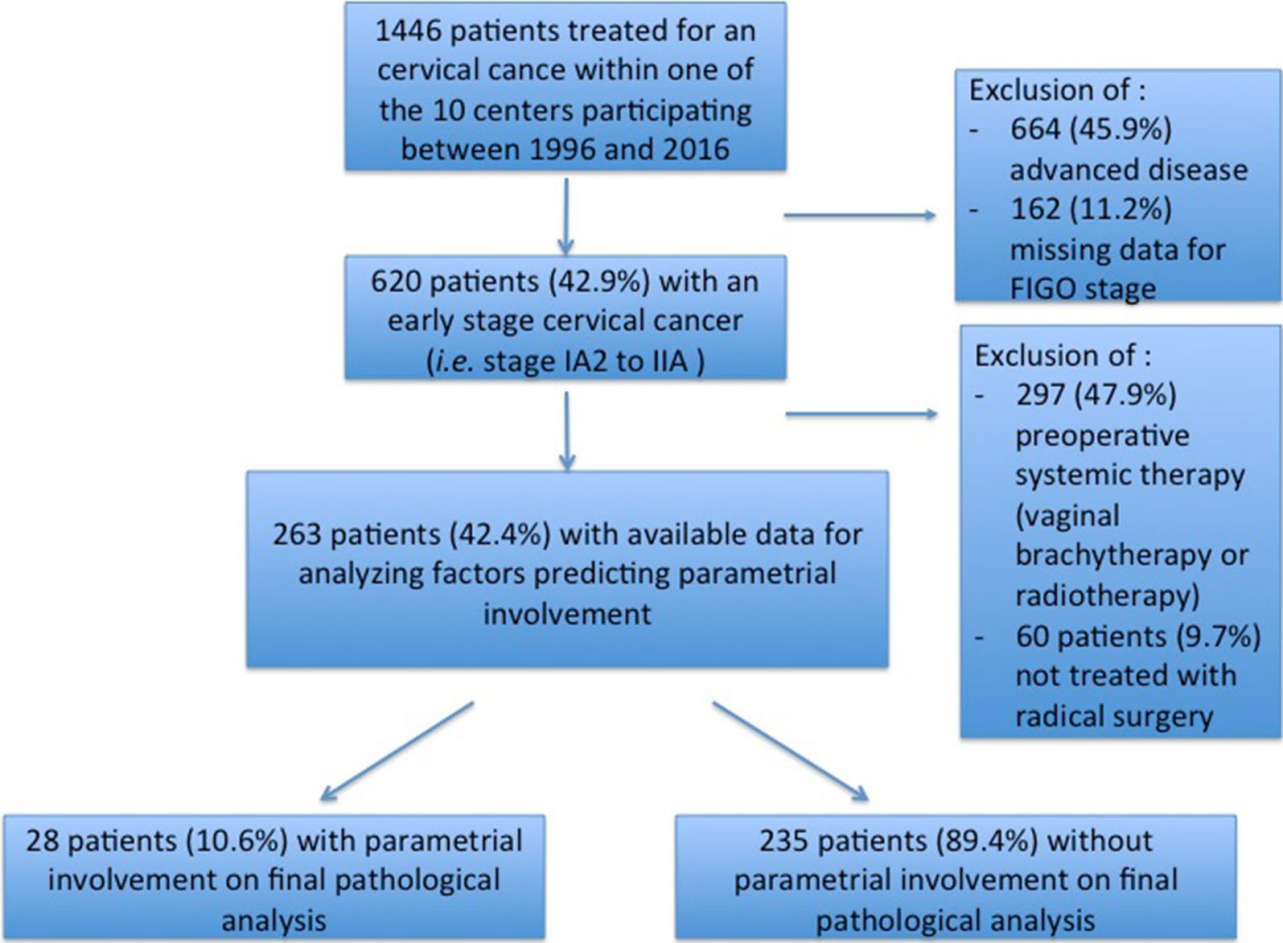
Patient flow chart

Between 1996 and 2016, 1446 patients were diagnosed and treated for a cervical cancer in the participating centers. Of these, 263 met our inclusion criteria and had data available for analysis as shown in the patient flow chart (Fig. 1). Mean follow up of the patients was 45.6 months (QI 17.4–64.0). Twenty-eight patients (10.6%) had parametrial invasion on final pathology analysis.

The preoperative clinical factors of the patients are presented in Table 1. Patients with parametrial invasion and those without were statistically different for most of the characteristics studied. In particular, patients with parametrial invasion were significantly older (p < 0.001), more often menopausal (p < 0.001), had a higher BMI (p < 0.01), and had more often disease staged IB2 or IIA (p < 0.001).

**Table 1:** Main characteristics of the included patients according to parametrial involvement

	Parametrial involvement n = 28 (10.6%)	No parametrial involvement n = 235 (89.4%)	p value
Age (years) (mean)	58.5 (IQ 46.2–72.5)	45.8 (IQ 36.5–52.5)	< 0.001
Menopausal			< 0.001
Yes	19 (67.9%)	67 (28.5%)	
No	9 (32.1%)	163 (69.4%)	
BMI (kg/m^2^) (mean)	26.2 (IQ 21–28.5)	24.8 (IQ 21–28.5)	< 0.01
Gestity (mean)	3.0	2.5	0.2
Parity (mean)	2.6	2.1	0.2
Pathologic type			0.1
Squamous	22 (78.6%)	154 (65.5%)	
Adenocarcinoma	3 (10.7%)	65 (27.7%)	
Others	3 (10.7%)	16 (6.8%)	
FIGO stage			0.001
IA	2 (7.1%)	28 (11.9%)	
IB1	16 (57%)	161 (68.5%)	
IB2	3 (10.7%)	4 (1.7%)	
IIA	5 (17.9%)	10 (4.3%)	

Table 2 summarizes the surgical management of patients with and without parametrial invasion. Patients with parametrial involvement were more likely to have undergone open surgery (p = 0.03) and less likely to have had surgical staging (p = 0.02). Patients with parametrial involvement also had significantly more peroperative complications [6 (21.4%) vs. 19 (8.1%), p = 0.03]. The rate of postoperative complications was similar in the two groups [6 (21.4%) vs. 41 (17.4%), p = 0.6] as was the rate of severe postoperative complications [3 (10.7%) vs. 12 (5.1%), p = 0.2]. Additional file 1 displays surgical outcomes by FIGO stages.

**Table 2:** Surgical outcomes and final pathologic analysis in patients with and without parametrial involvement

	Parametrial invasion	No parametrial invasion	p value
	n = 28 (10.6)	n = 235 (89.4)	
Surgical approach			0.02
Laparoscopy	16 (57.1)	141 (60)	
Laparotomy	10(35.7)	32 (13.6)	
Other (robotic, vaginal)	2 (7.1)	36 (15.3)	
Type of radical surgery			0.23
Hysterectomy	28 (100)	216 (91.9)	
Trachelectomy	0	19 (8.1)	
Pelvic and/or para-aortic lymphadenectomy	24 (85.7)	211 (89.8)	0.5
Total number of peroperative complications	6 (21.4)	19 (8.1)	0.03
Total number of postoperative complications	6 (21.4)	41 (17.4)	0.6
Number of severe postoperative complications (Clavien-Dindo ≥ 3)	3 (10.7)	12 (5.1)	0.2
Peritoneal cytology			< 0.001
Negative	18 (64.3)	214 (91.1)	
Positive	3 (10.7)	0	
Tumor size (mm)			
≤ 30	11 (39.3)	214 (91.1)	< 0.001
> 30	17 (60.7)	21 (8.9)	
Positive margins			< 0.001
Yes	11 (39.3)	6 (2.6)	
No	13 (46.4)	225 (95.7)	
Lymphovascular space invasion			< 0.001
Present	23 (82.1)	45 (19.1)	
Absent	4 (14.3)	190 (80.9)	
Lymph node involvement			< 0.001
Yes	8 (28.6)	18 (7.7)	
No	18 (64.3)	217 (92.3)	

The main pathologic features on final analysis were statistically different in the patients with and without parametrial involvement (Table 2). More specifically, patients with parametrial involvement had larger tumors (p < 0.001), and were more likely to have lymph node involvement (p < 0.001) and LVSI (p < 0.001).

### Survival analysis

**Fig. 2:**
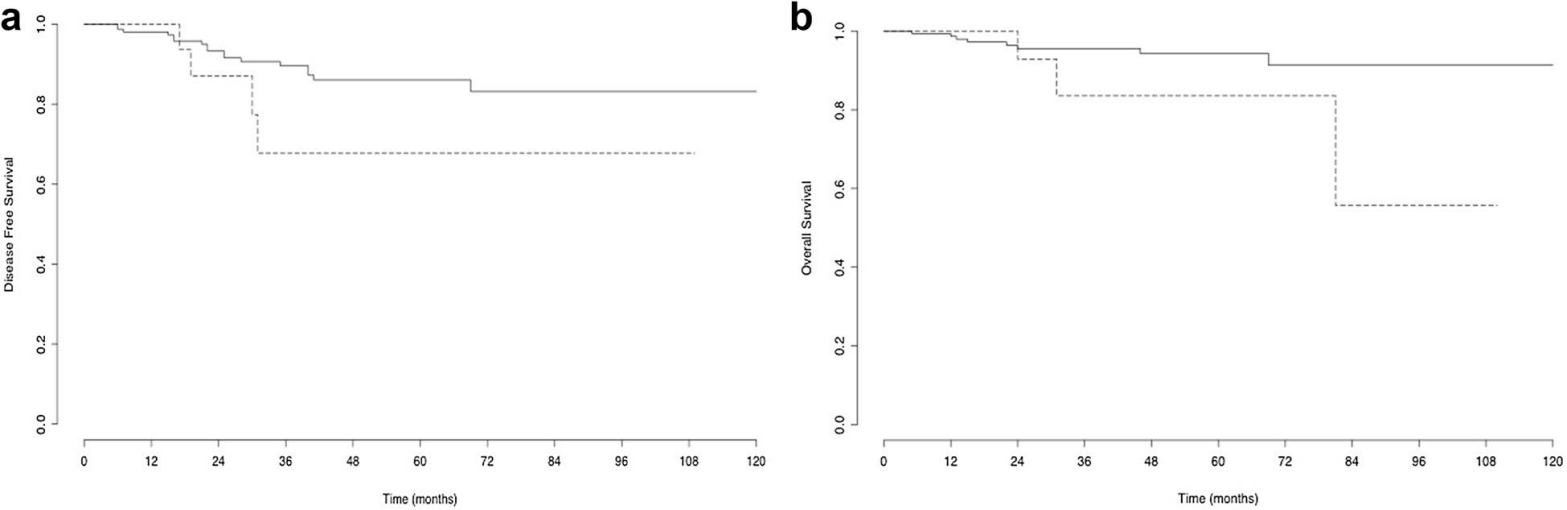
Kaplan–Meier curve for disease free survival (**a**) and overall survival (**b**) in patients with and without parametrial invasion on final pathologic analysis. Continued line is for patients without parametrial involvement. Dotted line is for patients with parametrial involvement (for DFS and OS, p = 0.153 and p = 0.08, respectively)

Patients with parametrial involvement had a lower OS (p = 0.08) (Fig. 2a) and DFS (p = 0.153) (Fig. 2b), without reaching statistical significance. All recurrences in patients with parametrial invasion (four patients) occurred within 3 years compared to 17/22 (77.3%) of those without parametrial invasion. Two of the patients with parametrial invasion who experienced recurrence had initial positive lymph nodes.

During follow up, three (10.7%) and 10 (4.25%) patients died in the group of patients with and without parametrial involvement, respectively. Mean OS was 41.4 (IQ 18–67) and 45.6 (IQ 17–62) months in patients with and without parametrial involvement, respectively.

### Predictive factors

An age at diagnosis of 65 years and tumor size of > 30 mm on preoperative MRI were the most relevant thresholds for predicting parametrial invasion, using an optimal threshold approach. In multivariate analysis, three clinicopathologic factors remained significantly associated with parametrial invasion: age > 65 years, a tumor > 30 mm and LVSI (Table 3). Our model showed an area under the receiver operating characteristic curve (AUC) of 0.95 with good calibration: the mean absolute error in predicted probabilities was 1.9%, and the maximum error was 11.8% with an unreliability index U < 0.0001 (Additional files 2, 3).

**Table 3:** Multivariate analysis for predictive factors associated with parametrial involvement on final pathologic analysis

Variable	Parametrial invasion		
	OR	CI (95%)	p value
Age at diagnosis	4.2	1.2–15.2	0.03
Menopausal status	3.5	0.9–13.8	0.07
BMI > 30	2.1	0.5–9.6	0.31
FIGO stage	1.9	0.4–9.4	0.44
Pathological type	0.7	0.3–1.8	0.43
Tumor size	8.6	2.8–26.3	< 0.001
Lymphovascular space invasion	13.4	3.7–48.2	< 0.0001
Positive pelvic lymph nodes	1.5	0.4–5.4	0.73

**Fig. 3:**
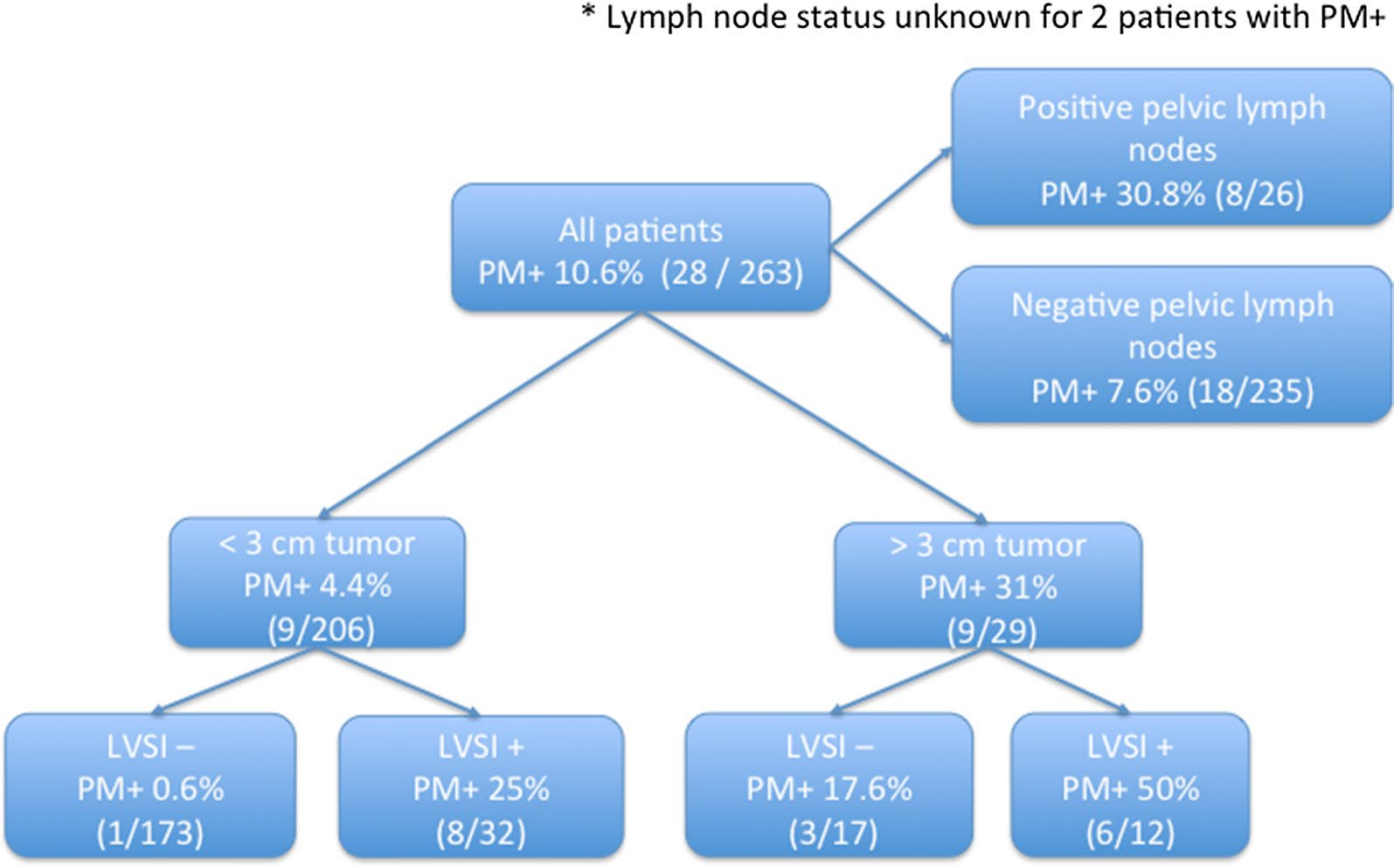
Flow chart of risk of parametrial involvement. *PM+/−* parametrial status, *LVSI* lymphovascular space invasion

**Fig. 4:**
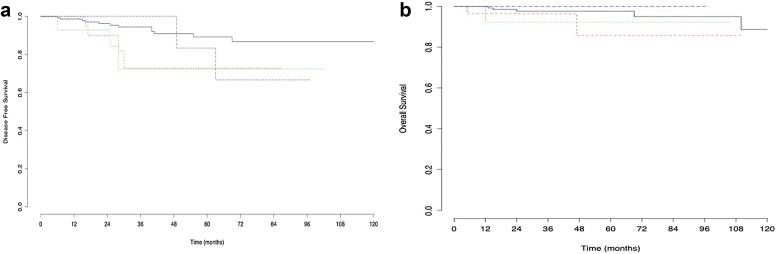
Kaplan–Meier curve for disease free survival (**a**) and overall survival (**b**) in patients stratified by tumor size (< or > 3 cm) and by LVSI status (positive or negative). In black, patients with tumors < 3 cm and without LVSI. In red, patients with tumors < 3 cm and LVSI. In green, patients with tumors > 3 cm and without LVSI. In blue, patients with tumors > 3 cm and LVSI

Parametrial disease was seen in 7.6% of the 235 patients with negative pelvic lymph nodes, compared with 30.8% of the 28 with positive pelvic nodes. The risk of parametrial invasion fell to 0.6% (1/173 patients) in the subgroup of women with negative pelvic nodes, tumors < 30 mm and no LVSI (Fig. 3). There was no significant difference in further subgroup analysis for DFS (p = 0.124) (Fig. 4a) or OS (p = 0.417) (Fig. 4b).

## Discussion

We report a large multicenter cohort of patients with early-stage cervical cancer treated with radical surgery and pelvic lymphadenectomy. Patients with parametrial invasion represented 11.9% of the patients included. Patients younger than 65 with tumors of less than 30 mm and without LVSI had a very low risk of parametrial invasion, especially in the node-negative group (0.6%).

Despite many studies addressing this issue, the role of parametrectomy in early-stage cervical cancer management remains controversial, mainly because of the morbidity associated with radical surgery. Urinary, sexual and anorectal dysfunction are well known direct consequences of parametrial resection [10, 12, 13, 25]. Reducing morbidity in radical surgery means either modifying the surgical technique or improving selection of the most eligible patients.

Pathology studies describing the tumor spread have failed to identify predictable patterns of dissemination with concomitant invasion of the medial and lateral parametria [26, 27]. Benedetti–Panici reported that parametrial invasion was through direct extension in 37% of cases, by lymph node metastases in 59% and LVSI in 52% [28]. These findings underline the difficulty of reducing the surgical extent of the resection without running the risk of leaving residual tumor tissue in situ.

Some authors have reported techniques leading to less morbidity, such as the “nerve sparing” hysterectomy described by Fuji et al. [29] or the “Laparoscopic Neuro-Navigation (LANN) technique” by Possover et al. [30]. However, even though these techniques show a benefit in terms of quality of life, they are difficult to perform in small volume centers and the learning curve limits their applicability in current practice, even if surgery of cervix cancer has to be performed by gynecologist oncologist in order to ensure its quality.

Another promising approach for predicting parametrial invasion is the nomogram. Nomograms have recently been developed to assess an individual probability of a certain event with validated indications in other gynecological malignancies [31, 32]. Kong et al. recently described such a nomogram for patients with stage IB disease [33]. Their model includes four parameters combining biological and imaging criteria to accurately predict parametrial invasion preoperatively. However, the model was developed in a population with a surprisingly high rate of parametrial invasion (64/298, 21.5%) when compared with what has been described in other studies including ours [34, 35]; especially as they only included patients with supposedly stage IB disease. Furthermore, the criteria they included are also somewhat surprising regarding the classic prognostic factors such as LVSI or tumor size, and may prove difficult to use in daily practice. Finally, external validation is required to test it.

Another way to reduce morbidity without compromising oncologic prognosis is through a better selection of patients that would benefit from radical surgery [36]. It would seem that there is a subgroup of patients among those with early-stage disease who are at very low risk of parametrial invasion. Most of the reports retain a tumor size < 2 cm, negative pelvic lymph nodes and the absence of LVSI, but there is currently a lack of consensus about these criteria [18, 37, 38]. It has been reported that elderly women have a higher incidence of LVSI and parametrial invasion even when their tumors are < 2–3 cm in size [20, 39]. This emphasizes the importance of including an age-based criterion to correctly identify patients at very low risk of parametrial invasion and is consistent with our findings.

Benedetti–Panici et al. identified parametrial lymph nodes in more than 90% of patients treated with radical hysterectomy for stage IIA or less cervical cancer [28]. Consequently, most authors concluded that the parametrium was the first “stop” on the tumor pathway to metastases. The development of the sentinel lymph node (SLN) procedure in early-stage cervical cancer, after being developed with great success in many other gynecological cancers, might challenge this model. Indeed, a recent study by Salvo et al. [40] found that only 4% of the SLNs were located within the parametrium. These results have been confirmed by others [41] with most SLNs being found in iliac or obturator locations. In our study, lymph node invasion was not significantly associated with parametrial invasion on multivariate analysis which is in line with the description of the SLN locations. Moreover, around half of the recurrences in patients with early-stage cancer after radical surgery are distant [42, 43]. This highlights our poor understanding of the factors associated with local and distant control and that we are still unable to properly identify patients who will benefit from surgery without experiencing high morbidity. In all fairness, we could suppose that generalization of the SLN approach would reduce morbidity to some extent as pelvic and paraaortic lymphadenectomy are partly responsible for the morbidity of radical surgery [44, 45].

From a patient’s perspective, it is of paramount importance to be able to determine the actual risk of parametrial invasion before performing morbid surgery. As many studies are currently evaluating less radical surgery in patients with early-stage cervical cancer [46], we feel these patients would highly benefit from a two-step approach: initial surgical staging and conization to assess LVSI and pelvic lymph node invasion followed by either a simple or radical hysterectomy/trachelectomy. The emergence of preoperative vaginal brachytherapy and neoadjuvant chemotherapy should increase the benefit harvested from such an approach by reducing the number of patients with parametrial invasion. Uzan et al. [47] reported that only one patient out of 162 had parametrial residual disease after preoperative vaginal brachytherapy. Similar results have been reported by other teams [48, 49]. Neoadjuvant chemotherapy response could also improve our selection of patients who might truly benefit from radical surgery as reported in a small cohort of 21 patients [50]. Unfortunately, our data were insufficient to further confirm these results.

Retrospective studies are often perceived as providing low-level evidence mostly because of missing data and patient selection bias. However, a major strength of our study lies in its multicentric nature and in the size of our cohort that could scarcely be reached using a prospective randomized trial, especially since parametrial invasion remains a rare event in early-stage cervical cancer. Our findings are perfectly in line with what has been reported elsewhere further confirming the need to adapt our management for early-stage cervical cancer patients by reconsidering the need for radical procedures for some patients. Finally, we eagerly await the results of three ongoing randomized controlled trials evaluating less radical surgery in patients with early-stage cervical cancer, such as the SHAPE and MD Anderson Centre studies [51–53].

## Conclusions

Our analysis suggests that there is a subgroup of patients with early-stage cervical cancer who do not benefit from radical surgery. These patients might be eligible for a two-step surgical approach consisting of initial nodal staging and conization prior to a radical procedure. This could reduce morbidity without jeopardizing oncological safety.

## Additional files

**Additional file 1: Table S1.** Surgical outcomes and final pathologic analysis in patients with and without parametrial involvement diversified by stage.

**Additional file 2.** Discrimination of the prediction model for predicting parametrial invasion in our cohort of 263 patients. Area under the curve: 0.95.

**Additional file 3.** Calibration of the prediction model for predicting parametrial invasion in our cohort of 263 patients. The x-axis represents the probability of parametrial invasion calculated with our model and y – axis represents the actual rate of parametrial invasion in our cohort.
