# Supplementary material for: Identification of a low risk population for parametrial invasion in patients with early-stage cervical cancer
Source: J Transl Med. 2018 Jun 14;16:163. doi: 10.1186/s12967-018-1531-6 (PMC6001133; doi:10.1186/s12967-018-1531-6)
Supplement: Supplementary file 1 — Additional file 1: Table S1. Surgical outcomes and final pathologic analysis in patients with and without parametrial involvement diversified by stage. [file 12967_2018_1531_MOESM1_ESM.docx]

|  | **IA (n = 30)** | **IB1 (n = 178)** | **IB2 (n = 7)** | **IIA (n = 15)** |
| --- | --- | --- | --- | --- |
| **Surgical approach** |  |  |  |  |
| *Laparoscopy* | *17 (56.7)* | *115 (64.6)* | *1 (14.3)* | *12 (80)* |
| *Laparotomy* | *4 (13.3)* | *30 (16.9)* | *4 (57.1)* | *0* |
| *Other (robotic, vaginal)* | *5 (16.7)* | *24 (13.5)* | *2 (28.6)* | *3 (20)* |
| **Type of radical surgery** |  |  |  |  |
| *Hysterectomy* | *23 (76.7)* | *170 (95.5)* | *7 (100)* | *15 (100)* |
| *Trachelectomy* | *7 (23.3)* | *8 (4.5)* | *0* | *0* |
| **Pelvic and /or para-aortic lymphadenectomy** | 19 (63.3) | 168 (94.4) | 7 (100) | 13 (80) |
| **Total number of peroperative complications** | *4 (13.3)* | *16 (9.0)* | *2 (28.6)* | *2 (13.3)* |
| **Total number of postoperative complications** | *2 (6.7)* | *42 (23.6)* | *2 (28.6)* | *1 (6.7)* |
| **Number of severe postoperative complications**  **(Clavien-Dindo** ≥**3)** | *0* | *13 (7.3)* | *2 (28.6)* | *1 (6.7)* |
| **Peritoneal cytology** |  |  |  |  |
| *Negative* | 26 (86.7) | 159 (89.3) | 3 (42.9) | 14 (93.3) |
| *Positive* | 1 (3.3) | 1 (0.6) | 1 (14.3) | 0 |
| **Tumor size** |  |  |  |  |
| *≤ 30 mm* | 28 (93.3) | 158 (88.8) | 0 | 8 (53.3) |
| *> 30 mm* | 2 (6.7) | 20 (11.2) | 7 (100) | 7 (46.7) |
| **Positive margins** |  |  |  |  |
| *Yes* | 2 (6.7) | 12 (6.7) | 1 (14.3) | 3 (20) |
| *No* | 26 (86.7) | 163 (91.6) | 4 (57.1) | 11 (73.3) |
| **Lymphovascular space invasion** |  |  |  |  |
| *Present* | 3 (10) | 49 (27.5) | 3 (42.9) | 7 (46.7) |
| *Absent* | 27 (90) | 128 (15.7) | 4 (57.1) | 8 (53.3) |
| **Lymph node involvement** |  |  |  |  |
| *Yes* | 0 | 18 (10.1) | 2 (28.6) | 5 (33.3) |
| *No* | 29 (96.7) | 160 (89.9) | 5 (71.4) | 10 (66.7) |
