# Supplementary figures and images for: Identification of a low risk population for parametrial invasion in patients with early-stage cervical cancer
Source: J Transl Med. 2018 Jun 14;16:163. doi: 10.1186/s12967-018-1531-6 (PMC6001133; doi:10.1186/s12967-018-1531-6)

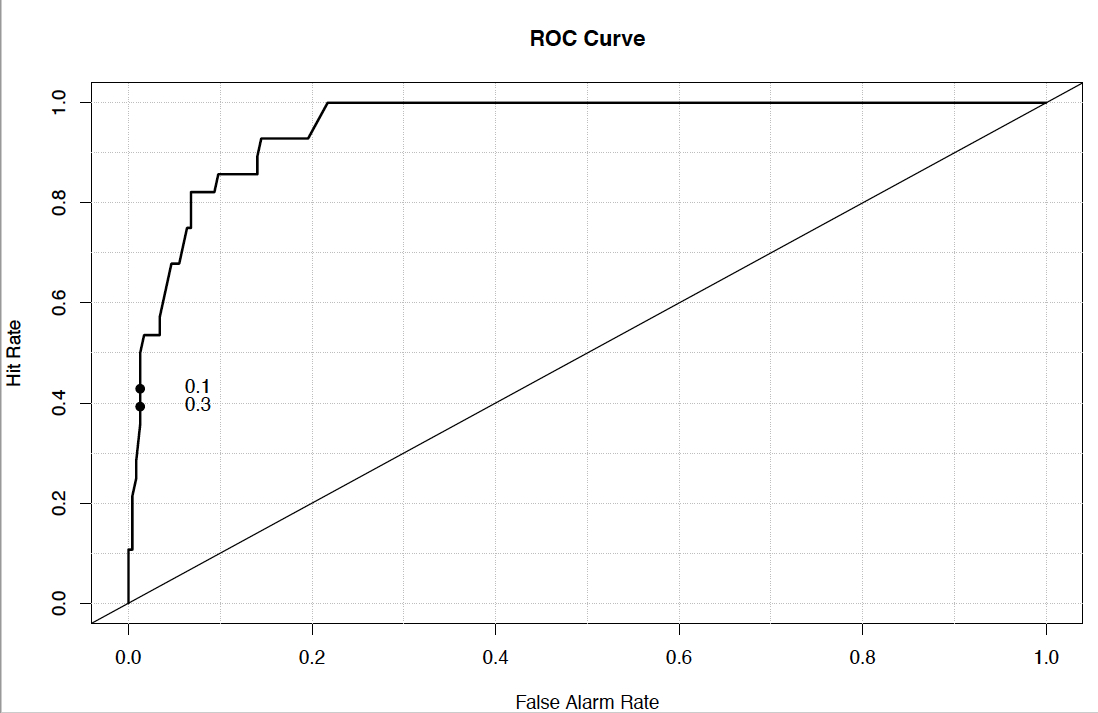

Supplement: Supplementary file 2 — Additional file 2. Discrimination of the prediction model for predicting parametrial invasion in our cohort of 263 patients. Area under the curve: 0.95. [file 12967_2018_1531_MOESM2_ESM.jpg]

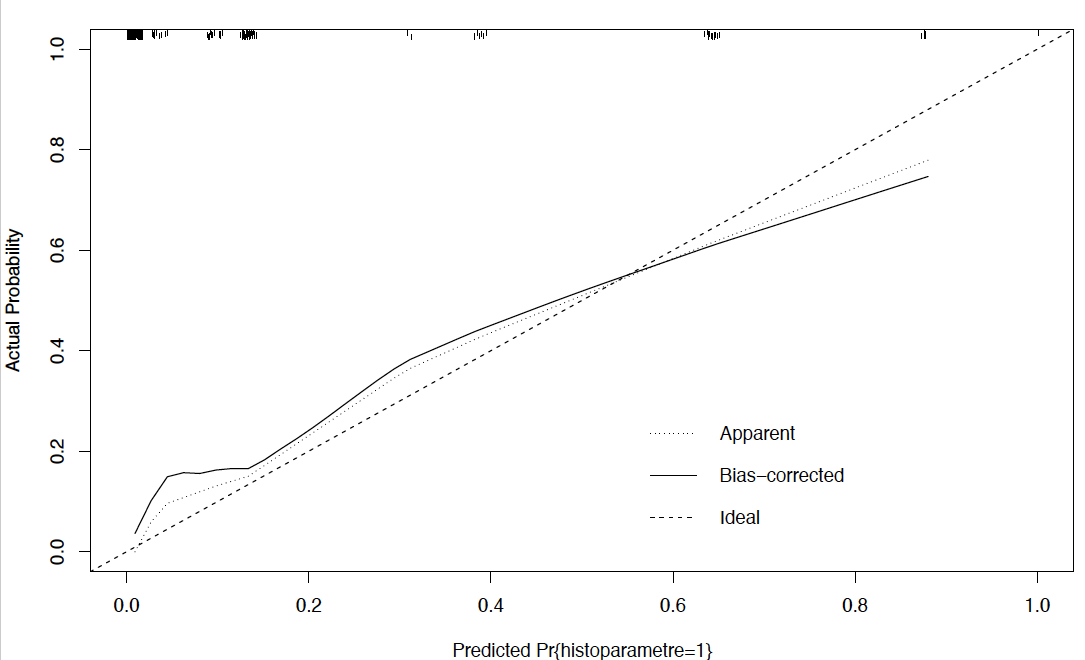

Supplement: Supplementary file 3 — Additional file 3. Calibration of the prediction model for predicting parametrial invasion in our cohort of 263 patients. The x-axis represents the probability of parametrial invasion calculated with our model and y – axis represents the actual rate of parametrial invasion in our cohort. [file 12967_2018_1531_MOESM3_ESM.jpg]
